# Building resistance: Stomatal and mesophyll conductance as variables limiting photosynthetic induction

**DOI:** 10.1093/plphys/kiaf199

**Published:** 2025-05-15

**Authors:** Pablo Ignacio Calzadilla

**Affiliations:** Assistant Features Editor, Plant Physiology, American Society of Plant Biologists; Institute for Integrative Biology of the Cell (I2BC), Université Paris-Saclay, CEA, CNRS Gif-sur-Yvette cedex 91198, France; Department of Earth and Environmental Sciences, Faculty of Science and Engineering, University of Manchester, Manchester M13 9PT, UK

Climate change has a negative impact on agriculture. The frequency of extreme weather events is increasing, hindering food production in a world with a growing population (Poppy et al. 2014). We need new crops with higher stress tolerance and yield, for which novel traits for improvement are required. Within the historically selected breeding traits, photosynthetic induction—the increase in photosynthetic rates when plants are exposed to increased light—has been largely unexplored despite its direct relationship with crop yield (Kromdijk and Long 2016). Studies addressing photosynthetic performance mainly focused on steady-state conditions of growth (e.g. control or stress). We need to understand the response of crops to unpredictable environments in which temperature, water availability, and light change dynamically. Limitations in photosynthetic induction can account for approximately 20% of the total photosynthetic capacity of leaves, leading to losses in carbon gain (Taylor and Long 2017; Eyland et al. 2021). By contrast, it has been shown that accelerating photosynthetic responses under fluctuating light can increase crop yield in the field (Kromdijk et al. 2016). As such, there is a gap in crop improvement through photosynthesis enhancement. Understanding the variables limiting this process can pave the way for improving crops by re-designing photosynthesis (Long et al. 2015).

In this issue of *Plant Physiology*, Pan et al. (2025) studied the factors limiting photosynthetic induction among historical cotton cultivars, mainly focusing on CO_2_ diffusion through the leaf and biochemical processes. The former can be divided into 2 steps: the diffusion of CO_2_ through the stomata into the leaf intercellular space (stomatal conductance, g_s_) and from the intercellular space into the carboxylation site within the chloroplast of mesophyll cells (mesophyll conductance, g_m_). Meanwhile, the maximum carboxylation rate (*V*_cmax_) accounts for biochemical limitations on carbon fixation (Figure). Although g_s_ and *V*_cmax_ are usually addressed in this type of study, the g_m_ contribution remains poorly known since the methods used for its estimation are expensive and not widely spread in the field of plant physiology. Nevertheless, g_m_ can significantly limit photosynthesis under fluctuating environments (Niinemets et al. 2009). Aiming to explore strategies for g_m_ estimation, Pan and colleagues first compare the well-established Δ^13^C method to the so-called fluorescence method (variable *J* method), which uses parameters measured through chlorophyll *a* fluorescence (Harley et al. 1992). No significant differences were observed when g_m_ was measured with both methodologies during the photosynthetic induction period of cotton, establishing the fluorescence method as a reliable technique to assess g_m_ in this crop. Thus, the latter was combined with gas exchange measurements to study the relative contribution of g_m_, g_s_, and *V*_cmax_ to photosynthetic induction.

Fourteen historical cotton cultivars were analyzed, showing different photosynthetic induction kinetics and non–steady-state photosynthetic rates (i.e. average photosynthetic rate over the first 5 and 15 min of induction, A_ave_5_min_ and A_ave_15-min_, respectively). No correlation was found between these parameters and the year of release of the cotton cultivars, although high among-cultivar variations suggest there are traits for positive selection. As demonstrated for other species (Koester et al. 2016), breeding did not aim to improve these traits in cotton, opening a gap for crop improvement in future selection efforts. By comparing the time to 90% induction (t_90_), authors identified g_s_ as the most significant factor limiting photosynthetic induction, followed by g_m_ and V_cmax_. Similar results were obtained by performing calculations of transient limitations over the entire induction period, where σ_stom_ accounts for approximately 67% of the limitation, while σ_mesophyll_ and σ_biochem_ account for 13% and 20%, respectively. These data show that g_m_ does not have a negligible impact on photosynthetic induction, and its lack of consideration will lead to inaccuracies when analyzing photosynthetic limitations (i.e. g_s_ and V_cmax_ contributions). Although g_s_, g_m_, and V_cmax_ will depend on species, cultivars, and the environmental conditions to which they are exposed, a deeper understanding of their relative contribution will allow the design of better strategies for crop improvement.

In conclusion, Pan et al. (2025) validated a reliable method to estimate mesophyll conductance using chlorophyll fluorescence, the latter being a widely available technique that can be used for studying photosynthetic induction. Using this methodology and gas exchange measurements, the authors successfully estimated the limitations of non–steady-state photosynthesis in cotton cultivars. Their data highlight the significance of stomatal conductance in limiting photosynthetic induction and the relevance of mesophyll conductance in photosynthetic responses. There is a gap between understanding photosynthetic physiology and identifying traits for crop improvement in a fluctuating environment. Pan and co-authors contribute to closing this gap by understanding photosynthetic induction.

**Figure.**
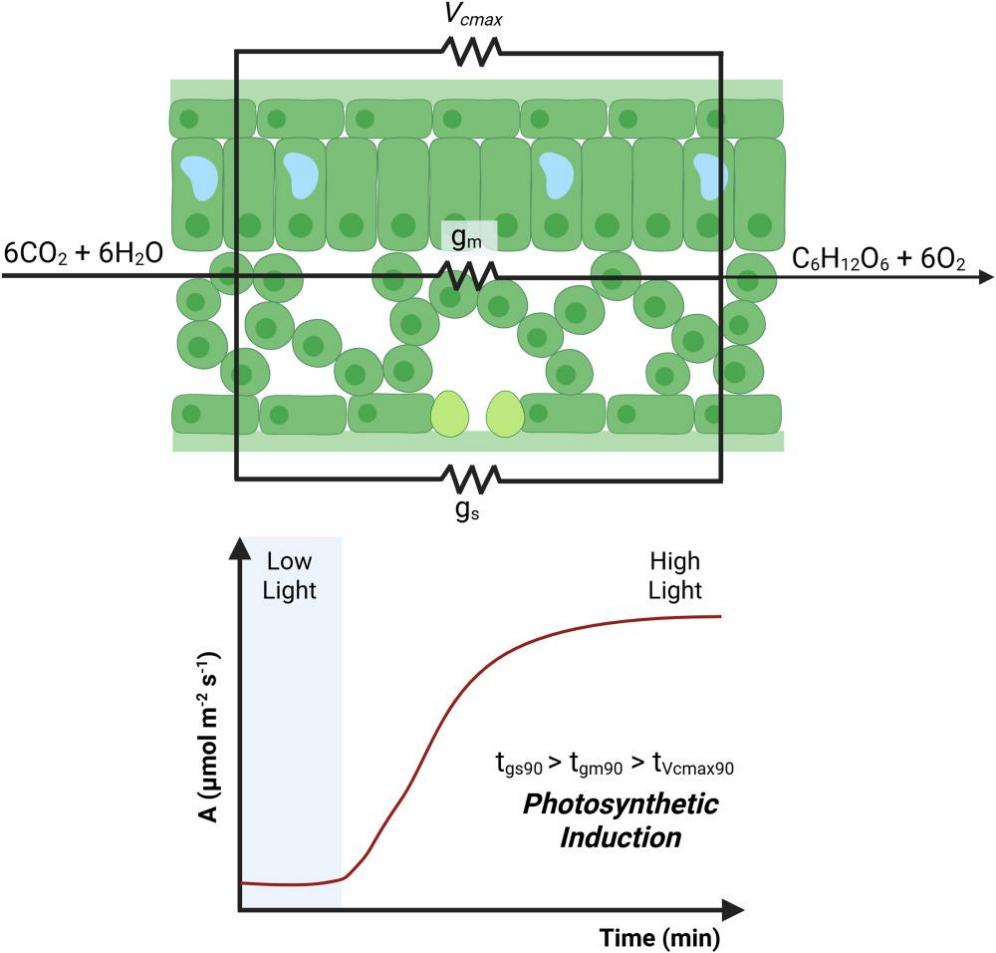
The g_s_, g_m_, and V_cmax_ limit photosynthetic induction in the transition from low to high light. By testing 14 historical cotton cultivars, Pan et al. (2025) identified g_s_ as the main contributor for this limitation (t_gs90_ > t_gm90_ > tV_cmax90_; t_90_ = time to 90% induction). Their data also highlight the relevance of g_m_ in this photosynthetic response, a factor that has not been widely addressed in non-steady state photosynthetic studies. g_m_ becomes significant for accurately understanding photosynthesis in a fluctuating environment. Created in BioRender. Calzadilla, P. (2025) https://BioRender.com/rbhjjxl.
